# A placebo-controlled efficacy study of the intravesical immunomodulators TMX-101 and TMX-202 in an orthotopic bladder cancer rat model

**DOI:** 10.1007/s00345-018-2334-3

**Published:** 2018-05-16

**Authors:** Johannes Falke, Christina A. Hulsbergen-van de Kaa, Roberto Maj, Egbert Oosterwijk, J. Alfred Witjes

**Affiliations:** 1 Department of Urology, Radboud University Nijmegen Medical Center, Geert Grooteplein Zuid 10 (610), P.O. Box 9101, 6500 HB Nijmegen, The Netherlands; 2 Department of Pathology, Radboud University Medical Center, Nijmegen, The Netherlands; 3 Telormedix SA, Bioggio, Switzerland

**Keywords:** Toll-like receptor, Non-muscle invasive bladder cancer, Imiquimod, TMX, Immunomodulator, TLR-7

## Abstract

**Purpose:**

TMX-101 and TMX-202 are formulations of toll-like receptor 7 (TLR-7) agonists, under investigation for the treatment of urothelial carcinoma. Our goal was to evaluate the efficacy of intravesical instillations of TMX-101 or TMX-202 in an orthotopic bladder cancer rat model.

**Methods:**

Four groups of 14 rats received an instillation with isogenic AY-27 tumor cells on day 0, starting tumor development. On day 2 and 5, the rats were treated with an intravesical instillation of TMX-101 0.1%, TMX-202 0.38%, vehicle solution or NaCl. On day 12 the rats were sacrificed and the bladders were evaluated histopathologically.

**Results:**

No signs of toxicity were seen. The number of tumor-positive rats was 11 of 14 (79%) in the vehicle control group and in the NaCl control group, versus 9 of 14 (64%) in the TMX-101-treated group, and 8 of 14 (57%) in the TMX-20-treated group. The difference between tumor-bearing rats in the treated and control groups was not significant (*p *= 0.12). Bladder weight was significantly lower for TMX-202-treated rats compared to vehicle (*p *= 0.005).

**Conclusions:**

TMX-101 and TMX-202 are TLR-7 agonists with antitumor activity. Treatment with TMX-101 and TMX-202 resulted in less tumor-bearing rats compared to vehicle or saline control groups, although not statistically significant. In this aggressive bladder cancer model, a lower number of tumor-positive rats after treatment with TLR-7 agonists indicates activity for the treatment of non-muscle invasive bladder cancer.

**Electronic supplementary material:**

The online version of this article (10.1007/s00345-018-2334-3) contains supplementary material, which is available to authorized users.

## Introduction

At any point in time, 2.7 million people have a history of urothelial bladder cancer worldwide [1]. Of the newly diagnosed cases, around 70% is non-muscle invasive bladder cancer (NMIBC) [2].

After transurethral resection of the bladder tumor (TURBT), for high-risk patients an adjuvant treatment with intravesical bacillus Calmette–Guerin (BCG) is recommended [2, 3]. BCG was the first successful immunotherapy for cancer and has been used for over 40 years to reduce the risk of bladder cancer recurrence as well as the risk of progression to muscle invasive disease.

Intravesical instillation of BCG induces a complex immune response leading to a Th-1 cell-mediated immune response responsible for cytotoxic T cell activity against urothelial carcinoma (UC) [4]. This cellular response depends on the Toll like receptor (TLR) signaling system [5]. TLR’s are a group of transmembrane proteins (TLR-1–10) expressed on immune cells, normal urothelium and urothelial carcinoma (UC) [6]. In BCG therapy, activation of TLR-2, 4 and 9 are involved [4, 7].

Despite being the gold-standard for adjuvant treatment, the 5-year recurrence rate is 41% in NMIBC patients treated with BCG for 1–3 years [8]. In addition, side effects of BCG therapy may occur ranging from mild local complaints in half of the patients, to severe systemic adverse effects, leading to cessation of treatment. Due to the modest success of the current treatments, new treatment options are under investigation.

The role of TLR’s in the antitumor activity of BCG supports further research on direct activation of TLR’s as potential therapy for UC. The immunomodulator imiquimod is a TLR-7 agonist widely used for the topical treatment of genital warts and (pre)malignant skin conditions [9, 10]. Application of imiquimod leads to induction of pro-inflammatory cytokines, and thereby to a strong antitumor cellular immune response via the MyD88-dependant pathway [11]. The therapeutic role of imiquimod may not be limited to the skin; studies show an inhibitory effect of imiquimod on UC [12]. To permit intravesical instillation of imiquimod, TMX-101 was developed. It shows an antitumor effect on bladder cancer cells, in vitro and in vivo [13, 14] and it has been tested for pharmacodynamics and pharmacokinetic in pigs [15] and rats [16], showing a good safety profile. Three clinical trials confirmed the safety and tolerability of TMX-101 [17–19]. TMX-202 is a highly specific agonist of TLR-7, which in in vitro cellular assays shows about two times stronger activity than imiquimod in inducing secretion of pro-inflammatory cytokines [13, 20]. Recently, we compared the pharmacokinetic and dynamic properties of TMX-202 and TMX-101 in the same F344 rat model showing a significant lower systemic uptake of TMX-202 compared to TMX-101 after intravesical administration of equimolar dose [16]. It may, therefore, have a better safety profile compared to TMX-101.

The goal of this study was to evaluate the efficacy of TMX-101 and TMX-202 compared to vehicle and saline in an orthotopic bladder cancer rat model.

## Materials and methods

### Tumor cells

The AY-27 rat bladder cancer cell line was established from a primary bladder tumor in *N*-[4-(5-nitro-2-furyl)-2-thiazolyl] formamide fed Fischer F344 rats. After thawing of the cells, they were passaged six times to revitalize the cells. Eventual passage numbers were 28 or 29. The cells were cultured as a monolayer in RPMI-1640 medium with l-glutamine (Invitrogen, Carlsbad, California), supplemented with 10% fetal calf serum (Sigma-Aldrich, St. Louis, MO), 100 U/mL penicillin-G and 100 μg/mL streptomycin (Invitrogen, Carlsbad, California) in a humidified 95% air/5% CO_2_-atmosphere. Medium was replaced two times a week, and when confluent, cells were split with standard trypsinisation procedures.

### Animals

A total of 56 female Fischer F344 rats (Charles River, L’Arbresle Cedex, France) were acclimatized for at least 1 week before start of the experiments. The rats, weighing 170 ± 10 g, were housed in cages (Techniplast, Milan, Italy) with goldflakes bedding (SPPS, Frasne, France) and environmental enrichment, in a temperature-controlled environment with a 12-hour light/dark cycle. Rats had free access to standard chow and water. Daily, the rats were weighed and monitored for well-being. Animal procedures were performed according to protocols, approved by the Institutional Animal Care and Use Committee (IACUC), committee for animal experiments (Radboud University Nijmegen Medical Centre, The Netherlands) and were in compliance with Dutch and European regulations. Group size was calculated using an α of 0.05, a power of 80 and 80% tumor development. The expected therapy effect was estimated to be 50% considering a pathological complete response (pT0) as primary endpoint. This resulted in a minimal group size of 14 rats.

### Tumor cell implantation

Tumor cell implantation was performed according to the protocol described by Xiao et al. [21]. Enrofloxacin (Bayer, Leverkusen, Germany) (5–10 mg/kg) was injected subcutaneously before catheterization. Experiments were performed under inhalation anesthesia: isoflurane 2–5% (induction), followed by isoflurane 2%, nitric oxide 0.5 L/min and oxygen 1 L/min. The bladder was catheterized via the urethra with a 16-gauge intravenous cannula (BD Biosystems, Erembodegem-Aalst, Belgium) and drained. The bladder was pre-conditioned with an instillation of 0.4 mL 0.1 M hydrochloride for 15 s and neutralized by adding 0.4 mL 0.1 M potassium hydroxide for again 15 s. The bladder was drained and flushed 3 times with 0.8 mL 0.01 M PBS. Within 30 min after harvesting, 1.5 × 10^6^ cells, resuspended in 0.5 ml medium, were instilled in the rat-bladder and left indwelling. After 1 h, the catheter was removed, and the rats could void spontaneously.

### Treatment

All rats received an intravesical instillation on day 2 and 5. The rats were anesthetized as described before. Subsequently, the rats were catheterized via the urethra, the bladder was drained and the pH was measured (pH indicator strips, Merck Milipore, Massachusetts, USA). Then, 0.5 ml instillation was administrated intravesically. Group 1 was treated with TMX-101 0.1% (4.16 mM). Group 2 was treated with TMX-202 0.38% (4.16 mM). Group 3 received an instillation with the vehicle (0.1 M lactic acid, poloxamer-407 16% and hydroxypropyl-β-cyclodextrin 5%) and group 4 received an instillation with NaCl as a control. Study drugs were provided by Telormedix SA (Switzerland), now part of UroGen Pharma Ltd. (Ra’anana, Israel). The instillation remained in the bladder for 1 h. After removal of the catheter, the pH of spontaneously voided urine was measured using pH-indicator strips.

### Pathological evaluation

On day 12, the rats were sacrificed using CO_2_ inhalation. At necropsy the internal organs were inspected and cystectomy was performed. The bladders were weighed, fixated using 4% buffered formalin (Boom B.V. Meppel, The Netherlands), laminated and embedded in paraffin. Sections of 5 μm were stained using hematoxylin and eosin (H&E). A specialized uro-pathologist (C.H.K.) blinded to the treatment groups, evaluated the bladder sections tumor stage according to the TNM-classification and tumor grade according to the 2004 WHO/ISUP classification. The invasion depth of the tumors was measured. The amount of inflammation in the bladder wall and/or surrounding tissue was scored 0 (no inflammation), 1 (mild), 2 (moderate) and 3 (severe inflammation). Data were analyzed using SPSS Statistics, Version 21.0 (IBM Corp., Armonk, USA). The graph was created using GraphPad Prism 5.03 (GraphPad Software Inc, USA).

## Results

During the experiment, there were no signs of impaired well-being of the rats. Mild hematuria on the day of catheterization was observed occasionally but on subsequent days, the urine returned normal. The pH of the urine before and after treatment showed no difference; the pH of all urines varied between 6.5 and 7.0. At necropsy no abnormalities to internal organs other than the bladder were seen. At macroscopic evaluation tumor-positive bladders appeared to have tumor mass without extravesical growth. Only one rat bladder (vehicle treated) showed a mass near the right ureteral orifice, extending towards the right ureter.

### Bladder weight

**Fig. 1:**
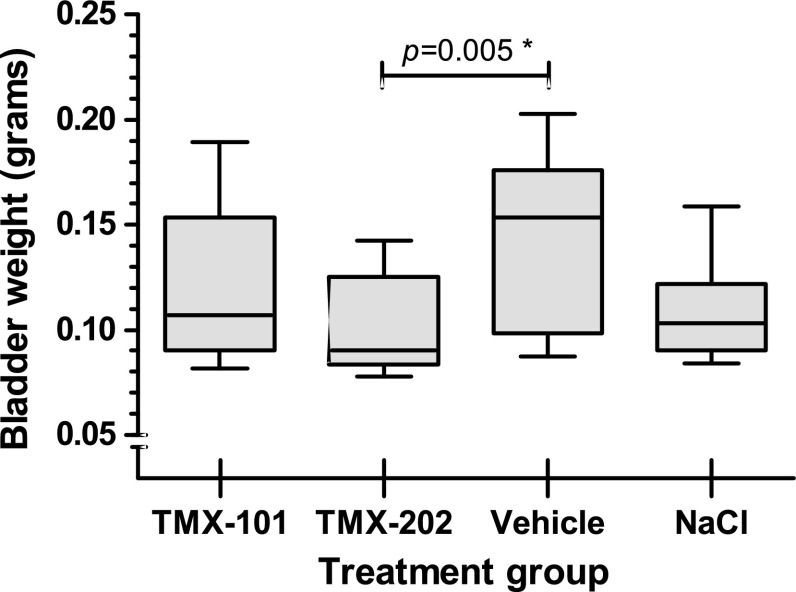
Boxplot showing the bladder weight in grams. Whiskers represent min. and max. values

The bladder weight, a substitute for tumor load is shown in Table 1. There was clear correlation between bladder weight and the presence of tumor (*p* < 0.0001, independent samples *T* test). The mean bladder weight of the TMX-202-treated animals was significantly lower compared to the vehicle group (*p* = 0.005, independent samples *T* test). No difference in mean bladder weight was seen between other groups. In Fig. 1, a boxplot shows the bladder weight per treatment group.

**Table 1:** Mean weight of the rat bladders per treatment group

Treatment group	Tumor	Mean bladder weight (g)	Standard deviation
TMX-101	Yes (*n* = 9)	0.137	0.032
	No (*n* = 5)	0.089	0.008
	All	0.120	0.035
TMX-202	Yes (*n* = 8)	0.114	0.022
	No (*n* = 6)	0.084	0.005
	All	0.101	0.023
Vehicle	Yes (*n* = 11)	0.162	0.030
	No (*n* = 3)	0.090	0.003
	All	0.144	0.042
NaCl	Yes (*n* = 11)	0.114	0.023
	No (*n* = 3)	0.088	0.004
	All	0.108	0.023
All	Yes (*n* = 39)	0.132	0.033
	No (*n* = 17)	0.088	0.006
	All	0.118	0.035

Weight in grams

### Inflammation

In almost all rat bladders, a certain degree of inflammation was present, showing infiltrating lymphocytes. Between groups, no statistically significant difference was observed (*p* = 0.106, Pearson’s Chi-square test). The mild and moderate degree of inflammation accounted for 87.5% of all 56 cases.

### Tumors and tumor response

The percentage of rats with urothelial carcinoma of the bladder was 64.3% (9/14) for the TMX-101 treated group, 57.1% (8/14) for the TMX-202-treated group, 78.6% (11/14) for the vehicle-control group and also 78.6% (11/14) for the NaCl-control group, see Table 2. In total, 69.6% (39/56) of the rats were tumor positive. All tumors show a pT2 stage, except one pTa tumor in a TMX-202-treated rat. The difference between tumor-negative rats in the treated and control groups was not statistically significant (*p* = 0.12 Fisher’s exact test). There was no significant difference between the individual groups in terms of tumor development. The treatment given was not predictive of the outcome (tumor positive versus tumor negative), when a logistic regression analysis was performed on the data; additional data on the logistic regression analysis can be found in Online resource 1.

**Table 2:** Number of rats with urothelial carcinoma of the bladder, per treatment group

Treatment group	Tumor free (%)	pTa (%)	pT2 (%)	Total (%)
TMX-101	5 (35.7)		9 (64.3)	14 (100)
TMX-202	6 (42.9)	1 (7.1)	7 (50.0)	14 (100)
Vehicle	3 (21.4)		11 (78.6)	14 (100)
NaCl	3 (21.4)		11 (78.6)	14 (100)

*pTa* non-invasive papillary carcinoma,* pT2* muscle-invasive urothelial carcinoma

### Invasion depth

Invasion depth of tumors was measured by the uro-pathologist (C.H.K.). The mean invasion depth measured was 1.33 mm for TMX-101, 1.42 mm for TMX-202, 1.50 mm for vehicle, and 1.39 mm for the NaCl group. The mean invasion depth of tumor-positive bladders did not show significant differences between individual treatment groups (independent samples *T* test, *p* = 0.486–0.912), or between TMX groups and control groups (independent samples *T* test *p* = 0.705).

## Discussion

The current standard of adjuvant treatment of high-risk UC is intravesical therapy with BCG. Despite common local and systemic side effects, BCG is still used widely because it is the superior treatment in reduction in risk of recurrence and progression [2, 3]. However, there is a need for drugs with less side effects, and alternative drugs in case of BCG unavailability [22]. As potential treatment options for urothelial carcinoma, the TLR-7 agonists TMX-101 and TMX-202 are subjects of research. The goal of the TLR-7 agonists TMX-101 and TMX-202 is a local immune response leading to antitumor activity compared to BCG, but circumventing the use of live attenuated mycobacteria, and thus preventing side effects. While normally being activated by ssRNA, research showed that TLR-7 can be activated equally by artificial ligands such as imiquimod [23]. Imiquimod is already successfully used as topical treatment for benign and malignant skin lesions [9, 10], but has also effect on urothelial carcinoma in vitro and in vivo [13, 14]. Hypothesizing that the bladder is accessible for ‘topical’ therapy, TMX-101 is a formulation of imiquimod for intravesical use and already tested in vivo for pharmacokinetics and pharmacodynamics [15] and in clinical trials [17–19]. TMX-101 is further developed under the brand name Vesimune by UroGen Pharma. TMX-202 is considered a stronger TLR-7 agonist compared to imiquimod [20], and may, therefore, have a stronger antitumor activity. Previously, we compared the pharmacodynamic and kinetic properties of TMX-101 and TMX-202 [16]. We showed a dose-dependent systemic uptake after intravesical instillation with a maximum plasma concentration of TMX-202 being 25 times lower compared to equimolar dosage of TMX-101. Stronger local activity combined with lower systemic uptake, may result in a favorable profile of TMX-202 compared to TMX-101.

The aim of this study was to evaluate the antitumor effects of intravesical TM-101 and TMX-202 compared to vehicle and saline control in an orthotopic and syngeneic bladder cancer rat model. After two instillations of TMX-101 or TMX-202, we observed more tumor-free rats compared to the vehicle or saline group; 35.7 and 42.9% versus 21.4 and 21.4%, respectively (not significant). One of the tumor-positive TMX-202-treated rats, however, showed a superficial pTa tumor in contrast to all other rats having pT2-tumors. This means that in the TMX-202 group 50% (7/14) rats were without invasive tumors. Still, in this study, the trend towards a higher number of tumor-negative rats in the treated groups compared to the control groups did not reach statistical significance. Because the observed therapy effect in our experiment was below the estimated 50%, the study was underpowered. Setting up this study, we estimated the therapy result based on in vivo studies of imiquimod on urothelial carcinoma [12, 24] and TMX-202 in a dermatological experiment [20].

There are, however, obvious differences between cutaneous application and an intravesical instillation with regard to duration of exposure. The TLR-7 agonists may need a longer duration of exposure to fully exert their role as immunomodulator. TLR-7 activation via the MyD88-pathway leads to transcription factors such as NK-κB and production of inflammatory cytokines, resulting in the recruitment of activated dendritic cells as well as other immune cells such as cytotoxic T cells [11, 25]. The treatment of malignant skin lesions by imiquimod may take up to several weeks. In addition, for intravesical therapy with BCG, it is known that the cellular response may take up to weeks to develop, but interestingly may persist for over a year after induction therapy [26]. Our experiment had a duration of 12 days with a total of two instillations, which may have been too short for optimal antitumor response. Rapid tumor development prevented a longer duration of our experiment.

The half-life of cutaneous imiquimod is between 21 and 27 h, with very limited systemic distribution [27]. It is easily applied, and treatment regimens vary between 3 and 7 applications a week with treatment duration up to 16 weeks [10, 27]. In contrast, intravesical exposure time of study drug is aimed at 1 h in the clinical situation. Prolonged retention times results in distention of the bladder and patients discomfort as well as dilution of the drug with urine. Additionally, intravesical instillation requires catheterization which is more burdensome than application on skin. Therefore, when compared to topical application, daily intravesical administration for weeks will likely not be tolerated by patients. Thus, if a TLR-7 agonist would have to be effective in a “standard” intravesical regimen of 6 weekly intravesical instillations followed by a maintenance scheme, it is of upmost importance that the receptor activation is as potent as possible. Since TMX-202 is considered to be up to 100 times more potent but with lower systemic uptake compared to imiquimod [20] this is a promising candidate for intravesical therapy.

Another limitation of our study is the model we used. The orthotopic rat bladder cancer model used in this study is considered a well-established bladder cancer model, but there are shortcomings. Tumors develop within 3–5 days, and muscle-invasive disease is observed in more than 50% of the rats after 6–7 days [28]. Besides an advanced tumor stage, the pathological grade is high grade (2004 WHO-classification) or grade 3 (1973 WHO-classification). Consequently, this model resembles high-grade muscle-invasive disease for which in clinical practice a cystectomy is recommended [2, 3]. Ideally, a bladder cancer animal model should have been used with a slow-developing tumor, with a long non-muscle invasive phase in which the agents are tested. Although we aimed for this early stage of tumor development with two instillations of study drugs on day 2 and 5, aggressive tumor development might have overtaken the treatment effect, resulting in the relative high percentage of tumor-positive rats in the treatment group.

In addition, pretreatment of the bladder may have caused an inflammatory response interfering with the TLR-7-induced response. Preconditioning the bladder with HCl is needed, as instilled urothelial cells require a damaged urothelial barrier for successful seeding [21, 28]. Although the urothelial barrier will restore rapidly [28], the inflammatory response is visible up to day 12 on histology. As there is no difference in degree of inflammation between treatment and control groups, this is likely a result of pretreatment. For future experiments, a control group without seeded tumor cells may be included, to isolate the inflammatory effect of TLR-7 activation.

Finally, the presence of a solid and bulky tumor, as seen in our experiment, may hamper both optimal TLR-7 activation as well as an efficient cellular response resulting in antitumor efficacy. A phase two study showed no ablative effect of TMX-101 after six instillations in patients with a low-grade pTa bladder tumor [19]. In the normal clinical situation, first a TURBT will be performed to resect all (or most of the) tumor. Additional intravesical therapy aims at a small residual tumor load, superficial *carcinoma* in situ and/or on preventing new tumor development and progression. A potential better efficacy in case of a low tumor load was suggested in a phase 2 pilot study, with TMX-101 a complete response was obtained in two of ten patients with carcinoma in situ after 6  weekly instillations [17].

Data on imiquimod and bladder cancer in the literature are limited. Two in vivo studies by the group of Scherr et al. showed a substantial decrease on tumor development after one [24] or two [12] intravesical instillations of imiquimod in the orthotopic bladder cancer C3H/JeJ mouse model [29]. In the first study, mice were treated 10 days after tumor cell implantation, and sacrificed at day 24. Four of 12 treated mice showed tumor, versus all 10 mice in the control group [24]. The other study describes intravesical treatment on day 1 and day 8 after tumor cell implantation, with sacrificing at day 15. Here, only 3 of 14 mice showed tumor versus 11 of 13 in the control group [12]. Although the model results in at least 85% invasive tumors, no data on tumor stage after treatment was given. These figures indicate a less aggressive model compared to the F344-AY27 rat model used in our study. In an in vivo mouse study, a significant reduction of tumor load (measured by bladder weight) is reported after three intravesical treatments with imiquimod, in C57BL/6 mice-bearing MB49 urothelial carcinoma [14]. In this study also no pathological results are reported. When bladder weight is used as surrogate marker for treatment effect, we do find a significantly lower bladder weight after TMX-202 treatment compared to vehicle, although this difference is not significant when compared to other treatment groups.

### Immunotherapy for NMIBC

Intravesical BCG for the treatment of bladder cancer is the gold standard for more than 40 years now and has proven to be the first successful immunotherapy against cancer. Apart from the TLR-7 agonists discussed here, recent and ongoing clinical trials reporting on immunotherapy for bladder cancer predominantly assess systemic checkpoint inhibitors (for example PD-L1, PD-1 and CTL-A4) targeting advanced and metastatic disease [30, 31]. However, the PD-1 inhibitor pembrolizumab is evaluated in combination with BCG for patients with NMIBC (NCT02808143 and NCT02324582). The PD-L1 antibody Atezolizumab is under investigation for the treatment of NMIBC (NCT02792192) as well as for patients with CIS (NCT02844816). Other investigational drugs aim to boost the immune response initiated by BCG, thereby hoping to improve its efficacy. For example, intravesical recombinant adenoviral Interferon-α2b [32] (NCT01687244) or the promising IL-15 superagonist complex ALT-803, that is administered intravesically in combination with BCG (NCT02138734). Out of the long list of promising immunotherapeutic targets [30, 31], hopefully soon an alternative to BCG will emerge, resulting in an improved disease management for patients suffering non-muscle invasive bladder cancer.

## Conclusions

In this in vivo experiment, we tested the TLR-7 agonists TMX-101 and TMX-202 in an orthotopic bladder cancer rat model. Treatment with two intravesical instillations of TMX-101 or TMX-202 resulted in less tumor-bearing rats compared to vehicle or saline control groups, although not statistically significant. This experiment turned out to be underpowered but in this aggressive bladder cancer model, a lower number of tumor-positive rats after treatment with TLR-7 agonists indicate a promising potential for the treatment of non-muscle invasive bladder cancer.

## Electronic supplementary material

Below is the link to the electronic supplementary material.

Supplementary material 1 (PDF 37 kb)
