# Supplementary material for: A placebo-controlled efficacy study of the intravesical immunomodulators TMX-101 and TMX-202 in an orthotopic bladder cancer rat model
Source: World J Urol. 2018 May 16;36(11):1719–25. doi: 10.1007/s00345-018-2334-3 (PMC6208681; doi:10.1007/s00345-018-2334-3)
Supplement: Supplementary file 1 — Supplementary material 1 (PDF 37 kb) [file 345_2018_2334_MOESM1_ESM.pdf]

World Journal of Urology 2018
Online Resource 1: Electronic Supplementary Material

A placebo controlled efficacy study of the intravesical immunomodulators TMX-101 and TMX-202 in an orthotopic bladder cancer rat model

Johannes Falke[1], Christina A. Hulsbergen-van de Kaa[2], Roberto Maj[3], Egbert Oosterwijk[1], J. Alfred Witjes[1]

[1]Radboud University Medical Center, Dept. of Urology, Nijmegen, the Netherlands
[2]Radboud University Medical Center, Dept. of Pathology, Nijmegen, the Netherlands
[3]Telormedix SA, Bioggio, Switzerland

Supplementary data
Logistic regression analysis: Predicting tumor outcome with treatment group.

Supplementary data: Table 1.

Observed and predicted frequencies for tumor presence by logistic regression with cutoff 0.5.

Observed		Predicted		
		Tumor		
		No	yes	% Correct
Tumor	No	0	17	0
	Yes	0	39	100
	Overall %			69.6

Supplementary data: Table 2.

Logistic regression analysis of 56 rats. Treatment group is not predictive of the outcome (tumor presence)

Treatment group	*p*	OR	95% CI
NaCl	*ref*		
TMX-101	0.41	0.49	0.09 - 2.63
TMX-202	0.23	0.36	0.07 - 1.91
Vehicle	1.00	1.00	0.16 - 6.08
Treatment overall	0.53		

OR: Odds Ratio, CI: Confidence Interval, ref: reference
